# Phytohormone and Transcriptomic Analysis Reveals Endogenous Cytokinins Affect Kiwifruit Growth under Restricted Carbon Supply

**DOI:** 10.3390/metabo10010023

**Published:** 2020-01-04

**Authors:** Simona Nardozza, Janine Cooney, Helen L. Boldingh, Katrin G. Hewitt, Tania Trower, Dan Jones, Amali H. Thrimawithana, Andrew C. Allan, Annette C. Richardson

**Affiliations:** 1 The New Zealand Institute for Plant and Food Research Limited (PFR), 1142 Auckland, New Zealand; dan.jones@plantandfood.co.nz (D.J.); amali.thrimawithana@plantandfood.co.nz (A.H.T.); andrew.allan@plantandfood.co.nz (A.C.A.); 2 The New Zealand Institute for Plant and Food Research Limited (PFR), 3240 Hamilton, New Zealand; janine.cooney@plantandfood.co.nz (J.C.); helen.boldingh@plantandfood.co.nz (H.L.B.); kati.hewitt@plantandfood.co.nz (K.G.H.); tania.trower@plantandfood.co.nz (T.T.); 3 School of Biological Sciences, University of Auckland, Private Bag 92019, 1142 Auckland, New Zealand; 4 The New Zealand Institute for Plant and Food Research Limited (PFR), 0294 Kerikeri, New Zealand; annette.richardson@plantandfood.co.nz

**Keywords:** cytokinin, fruit expansion, kiwifruit, phytohormone

## Abstract

Following cell division, fruit growth is characterized by both expansion through increases in cell volume and biomass accumulation in cells. Fruit growth is limited by carbon starvation; however, the mechanism controlling fruit growth under restricted carbohydrate supply is poorly understood. In a previous study using red-fleshed kiwifruit, we showed that long-term carbon starvation had detrimental effects on carbohydrate, anthocyanin metabolism, and fruit growth. To elucidate the mechanisms underlying the reduction in fruit growth during kiwifruit development, we integrated phytohormone profiling with transcriptomic and developmental datasets for fruit under high or low carbohydrate supplies. Phytohormone profiling of the outer pericarp tissue of kiwifruit showed a 6-fold reduction in total cytokinin concentrations in carbon-starved fruit, whilst other hormones were less affected. Principal component analysis visualised that cytokinin composition was distinct between fruit at 16 weeks after mid bloom, based on their carbohydrate supply status. Cytokinin biosynthetic genes (IPT, CYP735A) were significantly downregulated under carbon starvation, in agreement with the metabolite data. Several genes that code for expansins, proteins involved in cell wall loosening, were also downregulated under carbon starvation. In contrast to other fleshy fruits, our results suggest that cytokinins not only promote cell division, but also drive fruit cell expansion and growth in kiwifruit.

## 1. Introduction

*Actinidia* Lindl. spp. (kiwifruit) fruit growth and development is characterised by a rapid growth phase, where cell numbers in the ovary tissue rapidly increase by cell division (up to four weeks after full bloom [1] in the outer pericarp), and a cell expansion phase where cell volume increases and starch accumulates in cells [2]. Later in development, cell expansion and fruit growth slow down, with fruit progressing through maturity and ripening [3]. Carbohydrate supply manipulation of girdled canes (removal of a bark strip around the cane containing the phloem tissue) during *Actinidia chinensis* var. *deliciosa* ‘Hayward’ fruit development significantly increased fruit weight, whilst fruit weight was not affected in ungirdled canes due to the ability of kiwifruit to redistribute photosynthates within the vine [4]. In addition to ‘Hayward’, *A. chinensis* var. *chinensis* ‘Zes006′ fruit weight was also affected when carbohydrate supply was manipulated in girdled shoots [5,6,7].

The dynamic interplay of phytohormones regulates the growth and development of fleshy fruit [8]. Cell division is primarily driven by cytokinins, whilst auxins and gibberellins are major players in cell expansion [9,10]. Abscisic acid increases as the fruit approaches maturity and together with ethylene is responsible for fruit ripening [11]. Studies on endogenous hormone profiles during kiwifruit development are limited, with most of the studies focused on the effects of exogenous hormone applications on fruit growth [12,13]. In kiwifruit, increases in endogenous cytokinin concentrations have been associated with cell division [14], fruit maturity, and ripening [14,15]. It has also been shown that exogenous cytokinin applications to developing kiwifruit after cell division had ceased increased both cell and fruit expansions [12]. In plants, cell expansion is the result of turgor pressure stress caused by an increase in cell osmotic potential and cell wall relaxation mediated by low-pH activated expansin proteins [16]. Expansin genes have been reported to be amongst genes induced by cytokinin [17], but their role in cytokinin-driven growth has not been elucidated.

Sugar and hormone signalling networks have been extensively studied in vegetative tissues (reviewed by Ljung et al. [18]) and to a lesser extent in reproductive tissues [19]. Cytokinins, in particular, have been implicated in the control of source–sink relationships of carbon compounds [20], and a role for them in controlling the availability of sugars in sink tissues has been proposed [21,22]. In Arabidopsis, high quantities of photosynthetically generated sugars have an effect on plant growth and on de novo cytokinin biosynthesis through the upregulation of two genes coding for key enzymes catalysing active cytokinin precursors synthesis: isopentenyltransferase (*AtIPT3*) and cytokinin hydroxylase (*CYP735A2*) [23]. Arabidopsis type-B response regulators, part of the MYB transcription factor family, mediate cytokinin transcriptional regulation [24]. A meta-analysis of published transcriptomic data identified a number of primary metabolic genes that are regulated by cytokinins including trehalose 6-phosphate pathway genes [17] and the *bZIP11* transcription factor. These genes are involved in the growth regulatory network including the sucrose non-fermenting-1 related protein kinase 1, which controls carbohydrate metabolism [25]. 

Kiwifruit carbon starvation mainly occurs when the leaf-to-fruit ratio on vines is low, but it can also be caused by environmental factors such as high temperature or low light during critical fruit growth phases reducing the photosynthetic carbon supply to fruit. In this study, we hypothesized that fruit growth under restricted carbohydrate supply was controlled at the phytohormonal level. To test this, we integrated phytohormone profiles with transcriptomic and developmental datasets for fruit grown under high or low carbohydrate supplies. Of all the phytohormones, cytokinin concentration positively correlated with fruit weight and the carbohydrate supply treatment. Cytokinin biosynthetic genes were also significantly downregulated under carbon starvation. As several expansin genes were also significantly downregulated under carbon starvation, we propose that cytokinin signalling affects fruit weight via regulation of cell expansion through expansins.

## 2. Results

### 2.1. Fruit Weight and Phytohormone Correlations

**Figure 1:**
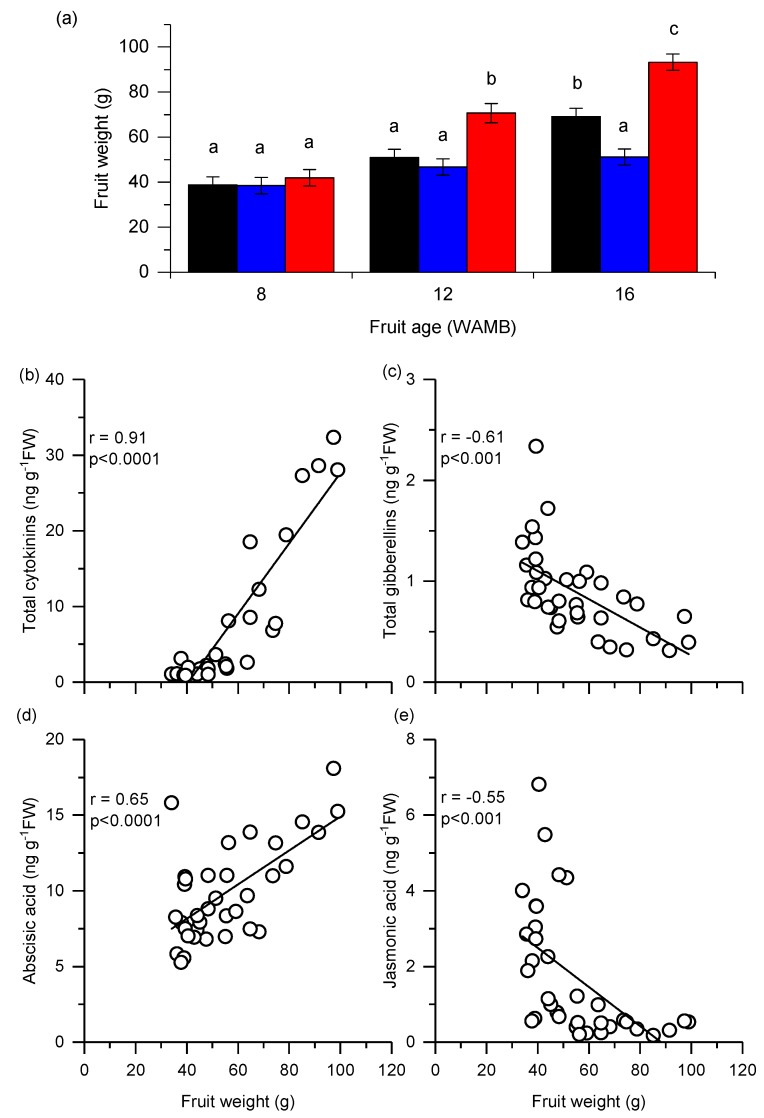
Fruit weight of kiwifruit (**a**) at three key stages of development: 8, 12, and 16 weeks after mid bloom (WAMB). Untreated control (black bar; vine standard), low carbohydrate supply (blue bar), and high carbohydrate supply (red bar). Values are averages ± SEM (*n* = 3 or 4). Statistical analysis by linear mixed effects model with type 3 sums of squares Kenward–Roger’s method; different letters mean a statistical difference, adjusted for multiple comparison by Tukey’s correction (*p* < 0.05). Pearson correlation analysis of between fruit weight and (**b**) total cytokinins, (**c**) total gibberellins, (**d**) abscisic acid, and (**e**) jasmonic acid concentration in fruit outer pericarp. *n* = 33 for cytokinins and *n* = 35 for the other phytohormones. FW, fresh weight.

Carbon starvation during fruit development significantly restricted kiwifruit fruit growth by 16 weeks after mid bloom (WAMB; Figure 1a). Low carbohydrate supply fruit (carbon starved) were 25% smaller than untreated control fruit from ungirdled shoots, and 45% smaller than high carbohydrate supply fruit.

Phytohormones from the following classes were quantified in kiwifruit tissues: cytokinins, gibberellins, auxins, jasmonates, salicylates, and abscisates.

To ascertain if a particular phytohormone class was correlated with fruit weight, Pearson correlation analysis was performed. Phytohormones were measured from the fruit outer pericarp as this is the major fruit tissue, representing 60% of fruit tissue proportion [26,27]. Fruit weight was positively and highly correlated with total cytokinin concentration (*r* = 0.91, *p* < 0.0001, Figure 1b). Fruit weight was also negatively and significantly correlated with total gibberellins (*r* = −0.61, *p* < 0.001, Figure 1c), positively and significantly correlated with abscisic acid (*r* = 0.65, *p* < 0.0001, Figure 1d), and negatively and significantly correlated with jasmonic acid (*r* = −0.55, *p* < 0.001, Figure 1e). Fresh weight was not significantly correlated with other phytohormones such as salicylic acid and auxin (data not shown).

### 2.2. Carbon Starvation Reduced Cytokinin Concentration in Fruit Outer Pericarp

**Figure 2:**
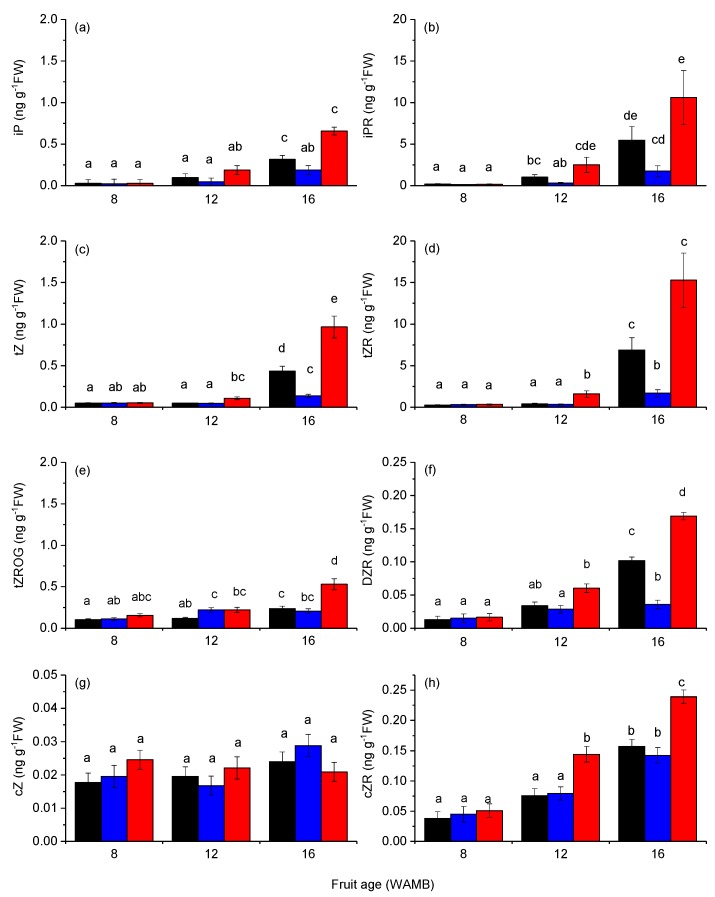
Cytokinin concentrations in the outer pericarp of developing kiwifruit: (**a**) iP, isopentenyl adenine; (**b**) iPR, isopentenyl adenine riboside; (**c**) tZ, *trans*-zeatin; (**d**) tZR, *trans*-zeatin riboside; (**e**) tZROG, *trans*-zeatin-*O*-glucoside; (**f**) DZR, dihydrozeatin riboside; (**g**) cZ, *cis*-zeatin; (**h**) cZR, *cis*-zeatin riboside.. Untreated control (black bar; vine standard), low carbohydrate supply (blue bar), and high carbohydrate supply (red bar). Values are averages ± SEM (*n* = 3 or 4). Statistical analysis by linear mixed effects model with type 3 sums of squares Kenward–Roger’s method; different letters mean a statistical difference adjusted for multiple comparison by Tukey’s correction (*p* < 0.05). Fruit age is in weeks after mid bloom (WAMB). FW, fresh weight.

The following cytokinin were quantified in kiwifruit outer pericarp tissue and were above the limit of detection (LOD): *trans*-zeatin (tZ), isopentenyl adenine (iP), *cis*-zeatin (cZ), dihydrozeatin riboside (DZR), isopentenyl adenine riboside (iPR), *trans*-zeatin riboside (tZR), *cis*-zeatin riboside (cZR), *trans*-zeatin-*O*-glucoside (tZROG), isopentenyl adenine-9-glucoside (iP9G), and *trans*-zeatin-9-glucoside (tZ9G) (Figure 2).

tZ and iP had similar concentrations and represented the major active cytokinins in developing kiwifruit, followed by cZ with a concentration that was one order of magnitude lower (Figure 2a,c,g). Dihydrozeatin (DZ) concentration was below the detection level. In untreated control fruit, the proportional concentration of active cytokinins to the total cytokinin pool decreased during development from 10% at 8 WAMB to 6% at 16 WAMB (Table S1). Cytokinin ribosides iPR and tZR were the major cytokinins (Figure 2b,d). Other ribosides DZR and cZR were also detected, but their concentrations were one order of magnitude lower than those of iPR and tZR (Figure 2f,h). In untreated control fruit, the proportional concentration of cytokinin ribosides increased during development from 50% at 8 WAMB to 90% at 16 WAMB (Table S1), and this pattern was shared with the other two treatments. For the reversible *O*-glycosylated conjugates, only tZROG was above the detection level and its proportional concentration decreased in untreated control fruit from 10% at 8 WAMB to 2% at 16 WAMB (Table S1). Irreversibly *N*-glycosylated cytokinins (iP9G and tZ9G) also decreased in untreated control fruit during development, from 25% at 8 WAMB to 2% at 16 WAMB (Table S1). By 16 WAMB, carbon starvation significantly reduced the majority of the active cytokinins and their metabolic or glycosylated intermediates (Figure 2). iP and its riboside iPR were significantly lower in carbon-starved fruit (low carbohydrate supply; Figure 2a,b). Carbon starvation had a similar effect on tZ, tZR, and tZROG, and their concentrations were significantly lower in carbon-starved fruit than in high carbohydrate supply fruit (Figure 2c,d,e). DZR concentration was also significantly lowered by carbon starvation (Figure 2f). cZ was not affected by carbon starvation, but cZR concentrations were significantly higher in high carbohydrate supply fruit than in carbon-starved fruit and the untreated control (Figure 2g,h).

**Figure 3:**
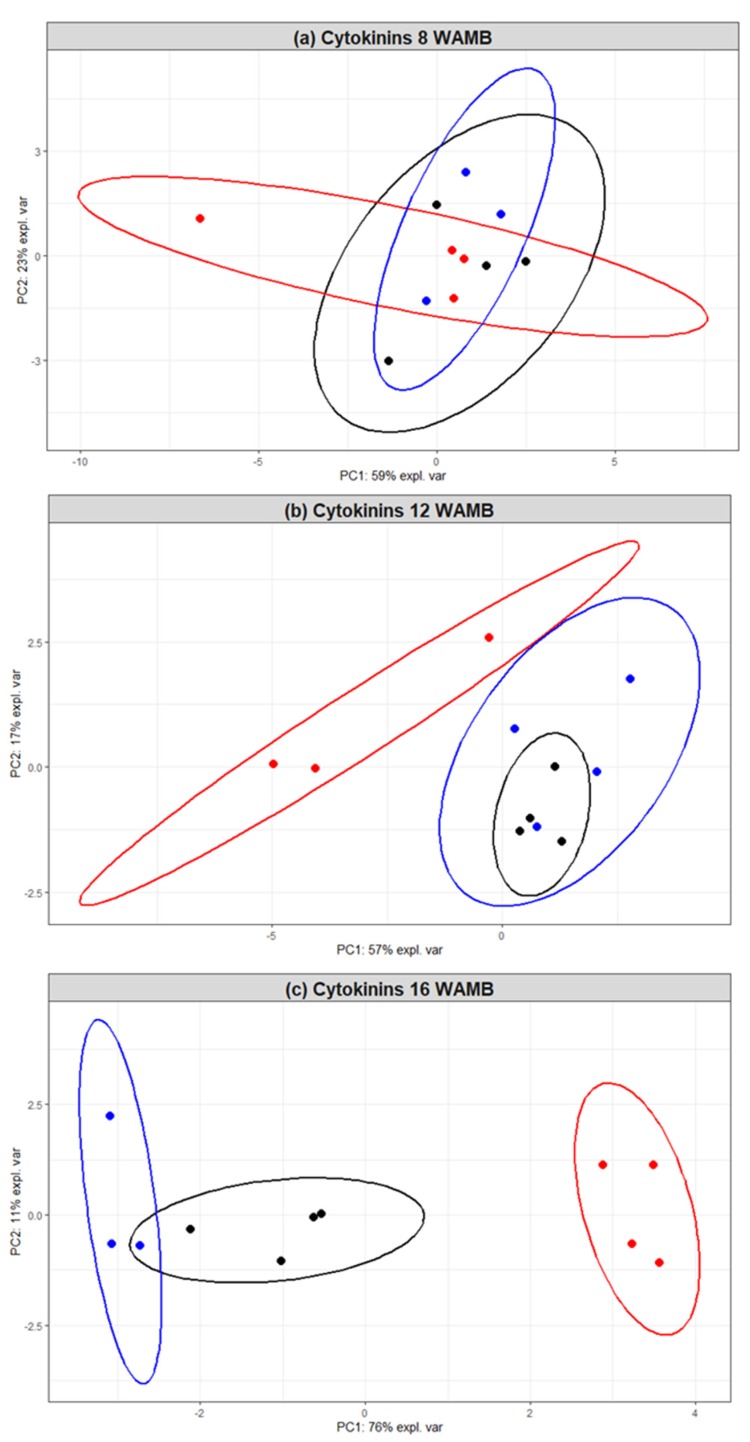
Principal component analysis (PCA) based on cytokinin concentrations in the outer pericarp of developing kiwifruit: (**a**) 8 weeks after mid bloom (WAMB); (**b**) 12 WAMB; (**c**) 16 WAMB. Black, untreated control (vine standard); blue, low carbohydrate supply (carbon starvation); red, high carbohydrate supply. PC, principal component. Confidence ellipses: *p* < 0.05.

We further employed principal component analysis to assess the effects of the treatment on cytokinin composition. It is shown in Figure 3 that 59%, 57%, and 76% of variation can be explained by principal component 1 (PC1) at 8, 12, and 16 WAMB, respectively. An effect was first observed at 12 WAMB, with the cytokinin composition of high carbohydrate supply treatment fruit being distinct from that of the untreated control fruit and low carbohydrate supply treatment. At 16 WAMB, the data clustered into three distinct cohorts, of which high and low carbohydrate supply treated fruit showed the highest divergence in cytokinin composition. Dimension 1 was strongly and positively driven by all the measured cytokinins, with the exception of cZ, which mainly drove dimension 2 (Figure S1).

### 2.3. Carbon Starvation Had Lesser Effects on Other Phytohormones

**Figure 4:**
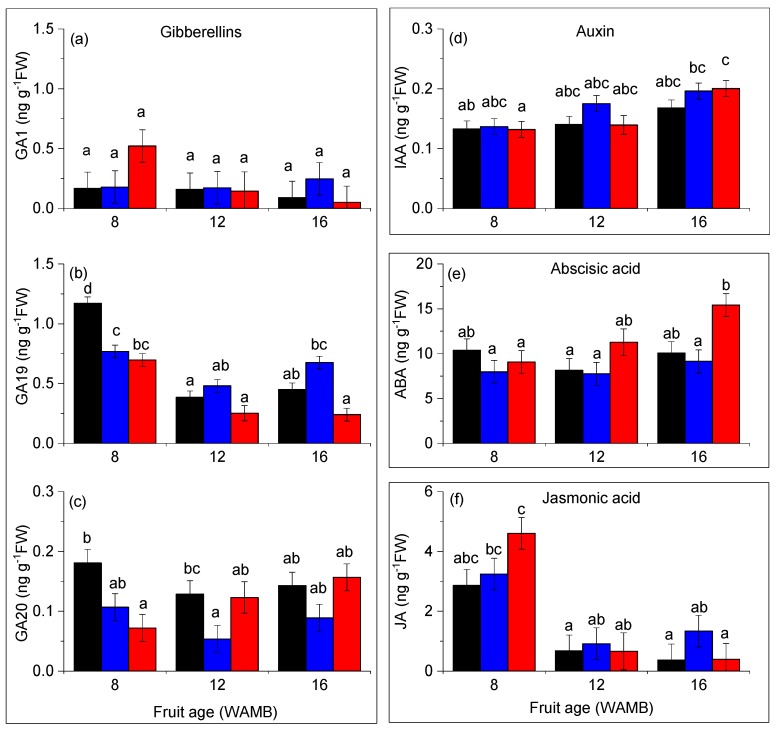
Gibberellins, auxin, abscisic acid, and jasmonic acid concentrations in the outer pericarp of developing kiwifruit: (**a**) GA1, gibberellin A1; (**b**) GA19, gibberellin A19; (**c**) GA20, gibberellin A20; (**d**) IAA, indole-3-acetic acid; (**e**) ABA, abscisic acid; (**f**) JA, jasmonic acid.. Untreated control (black bar; vine standard), low carbohydrate supply (carbon starvation; blue bar), and high carbohydrate supply (red bar). Values are averages ± SEM (*n* = 4). Statistical analysis by linear mixed effects model with type 3 sums of squares Kenward–Roger’s method; different letters mean a statistical difference, adjusted for multiple comparison by Tukey’s correction (*p* < 0.05). Fruit age is in weeks after mid bloom (WAMB). FW, fresh weight.

Gibberellins GA1, GA19, and GA20 were present in the outer pericarp of kiwifruit in concentrations above the limit of detection (Figure 4a–c). Active gibberellin GA1 concentrations were 4-fold lower than concentrations of its precursor GA19. GA19 was the most abundant gibberellin, and its concentration decreased 2-fold during fruit development. GA20 was present at 4- to 8-fold lower concentrations than GA19, and its concentrations were stable during development. Under carbon starvation, GA19 concentrations decreased less during fruit development than in the other treatments (Figure 4b), and at 16 WAMB, it was about 3-fold higher than in high carbohydrate supply fruit (*p* < 0.0001). 

Indole-3-acetic acid (IAA) was the only auxin detected by our system and its concentration slightly increased during fruit development (Figure 4d). Carbon starvation had no effect on IAA concentrations.

The concentration of the stress hormone abscisic acid (ABA) was stable during fruit development, although by 16 WAMB, its concentration in high carbohydrate supply fruit was significantly higher than in fruit from the other treatments (Figure 4e). This suggests that high carbohydrate supply fruit was the main driver of the positive correlation between fruit weight and ABA observed in Figure 1d (data not show). Jasmonic acid (JA) concentration decreased during fruit development and at 8 WAMB, its concentration was significantly higher in high carbohydrate supply fruit than in fruit from the other treatments (Figure 4f). JA bioactive isoleucine conjugate (JA-Ile) was below the detection level.

### 2.4. Carbon Starvation Downregulates Cytokinin Biosynthetic Genes

**Figure 5:**
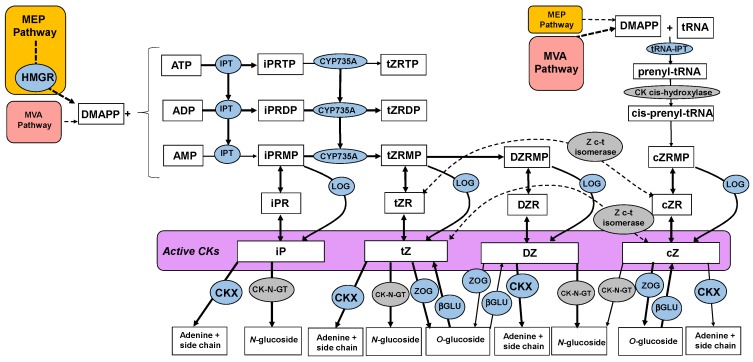
Cytokinin biosynthetic and catabolic pathways. Enzymes in blue are known and have been identified in kiwifruit; enzymes in grey are unknown and/or not identified in kiwifruit. Modified from [28,29]. βGLU, β-glucosidase; CK, cytokinin; CK-N-GT, cytokinin *N*-glucosyltransferase; CKX, cytokinin dehydrogenase; CYP735A, cytokinin hydroxylase; cZ, *cis*-zeatin; cZR, cZ riboside; cZRM(/D/T)P, cZ nucleotides; DMAPP, dimethylallyl pyrophosphate; DZ, dihydrozeatin; DZR, DZ, riboside; DZRM(/D/T)P, DZ nucleotides; HMGR, 3-hydroxy-3-methylglutaryl-coenzyme A reductase; iP, *N*^6^-(Δ^2^-isopentenyl)adenine; iPR, iP riboside; iPRM(/D/T)P, iP nucleotides; IPT, adenosine phosphate-isopentenyltransferase; MEP, methylerythritol phosphate pathway; LOG, LONELY GUY (cytokinin riboside 5’-monophosphate phosphoribohydrolase); MVA, mevalonate pathway; tRNA, transfer RNA; tZ, *trans*-zeatin; tZR, tZ riboside; tZRM(/D/T)P, tZ nucleotides; ZOG, zeatin *O*-glucosyltransferase.

The cytokinin biosynthetic and catabolic pathway was constructed (Figure 5) using published information [28,29]. Gene models that code for the enzymes in the cytokinin pathway were identified by gene mining the kiwifruit genome [30] on the basis of previously published kiwifruit data [15] and homologues from other species [31,32], marked as blue in the chart (Figure 5). Where genes coding for a particular enzyme were unknown in Arabidopsis or were not found for kiwifruit, the enzymes were marked in grey.

**Figure 6:**
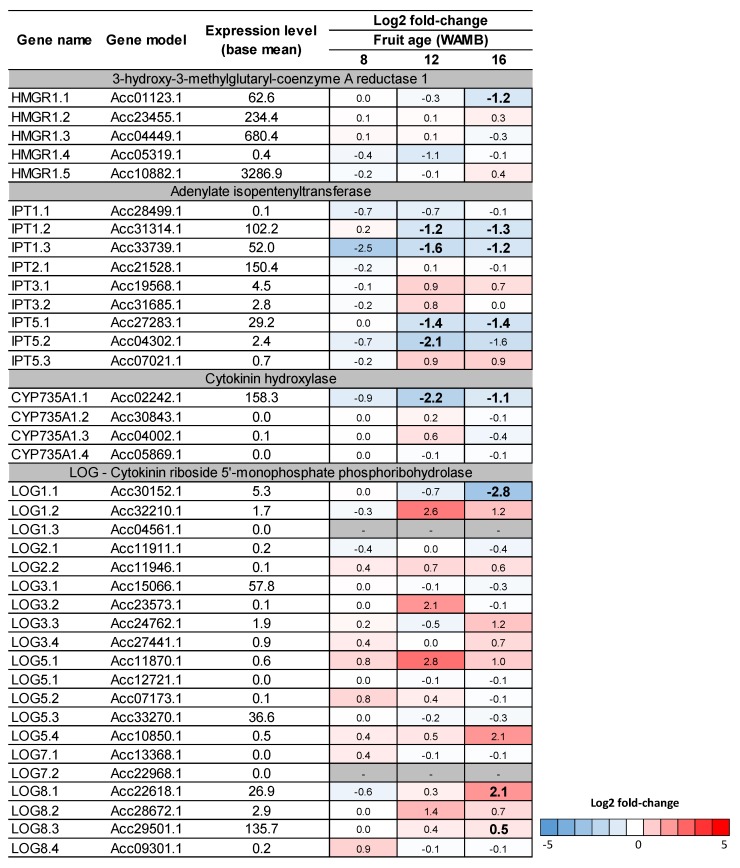
Heat-map of kiwifruit cytokinin biosynthetic genes. DESeq comparison of low carbohydrate supply (carbon starvation) versus high carbohydrate supply treatments. Differentially expressed genes were identified based on the DESeq analysis from Nardozza et al. [6]. For each gene, the expression level (base mean) and the log2 fold-change are presented. Fruit age is in weeks after mid bloom (WAMB). Bold figures mean differences are significant for adjusted *p* < 0.05 (DESeq analysis).

The expression of a number of genes involved in cytokinin biosynthesis was reduced under carbon starvation. Five genes of the 3-hydroxy-3-methylglutaryl-coenzyme A reductase 1 family were expressed in kiwifruit and the *HMGR1.1* gene was significantly downregulated under carbon starvation (Figure 6). We identified nine adenylate isopentenyltransferase (*IPT*) genes: three *IPT1*, one *IPT2*, two *IPT3*, and three *IPT5*. Of these, *IPT1.2*, *IPT1.3*, and *IPT5.1* were significantly downregulated under carbon starvation from 12 WAMB. Of the four cytokinin hydroxylase 1 genes (*CYP735A1*) expressed, *CYP735A1.1* was significantly downregulated under carbon starvation from 12 WAMB.

**Figure 7:**
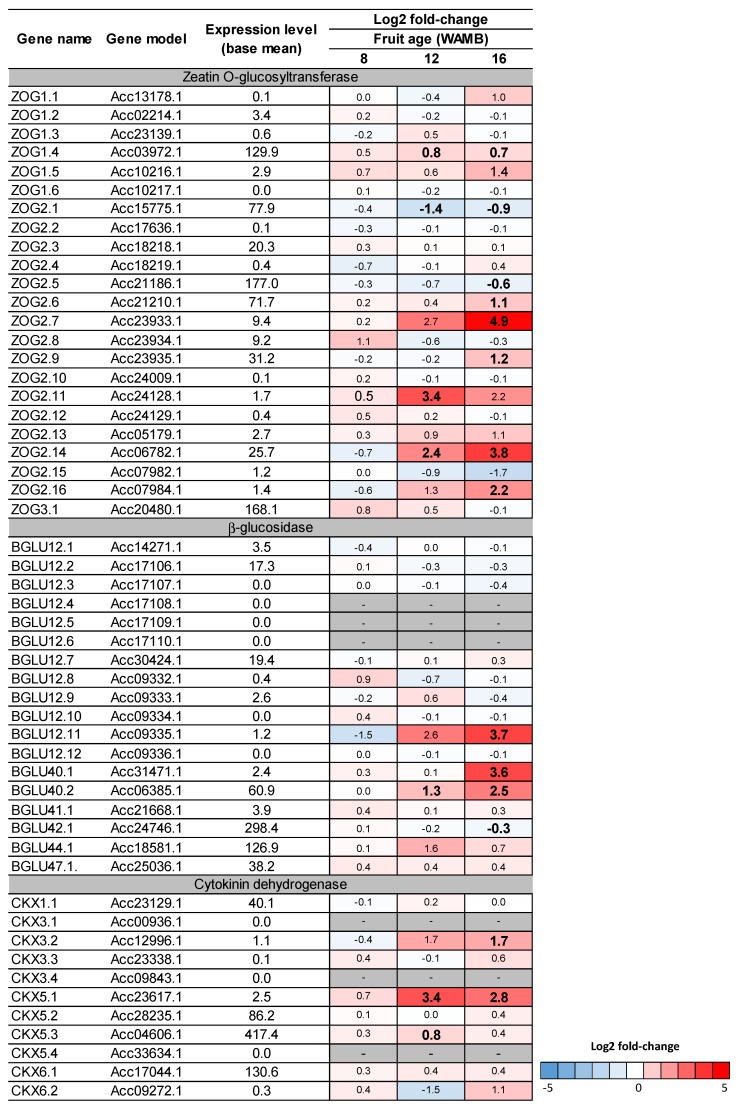
Heat-map of kiwifruit cytokinin catabolic genes. DESeq comparison of low carbohydrate supply (carbon starvation) versus high carbohydrate supply treatments. Differentially expressed genes were identified based on the DESeq analysis from Nardozza et al. [6]. For each gene, the expression level (base mean) and the log2 fold-change are presented. Fruit age is in weeks after mid bloom (WAMB). Bold figures mean differences are significant for adjusted *p* < 0.05 (DESeq analysis).

Expression of cytokinin catabolic genes were also affected by carbon starvation, although to a lesser extent than the biosynthetic genes, with an overall upregulation of the genes involved in reversible conjugation (zeatin *O*-glucosyltransferase, ZOG; β-glucosidase, βGLU) or irreversible cleavage (cytokinin hydrogenase, CKX) (Figure 7). The main cytokinin dehydrogenase (*CKX5.3*) was only significantly upregulated at 12 WAMB.

### 2.5. Carbon Starvation Effects on the Genes in the Multistep Phosphorelay (MSP) Cytokinin Signalling

The genes involved in the first two steps of the multistep phosphorelay (MSP) cytokinin signalling were upregulated by carbon starvation (Figure S2). Histidine kinases *AHK2.1*, *AHK3.5*, *AHK4.2*, and *AHK4.3* were significantly upregulated at 16 WAMB (2-, 1.8-, 1.6-, and 1.6-fold, respectively). The histidine-containing phosphotransfer protein *AHP1.7* was 5-fold upregulated under carbon starvation. Four of the 12 A-type Arabidopsis Response Regulators (type-A ARR) genes identified in kiwifruit, which are negative regulators of cytokinin signalling, were downregulated under carbon starvation whilst four of the 19 type-B ARRs genes, which act as positive regulators of cytokinin signalling, were upregulated by this treatment (Figure S3). *ARR12.5* was 1.5-fold upregulated by carbon starvation from 12 WAMB.

### 2.6. Carbon Starvation Results in Downregulation of Expansin Genes

**Figure 8:**
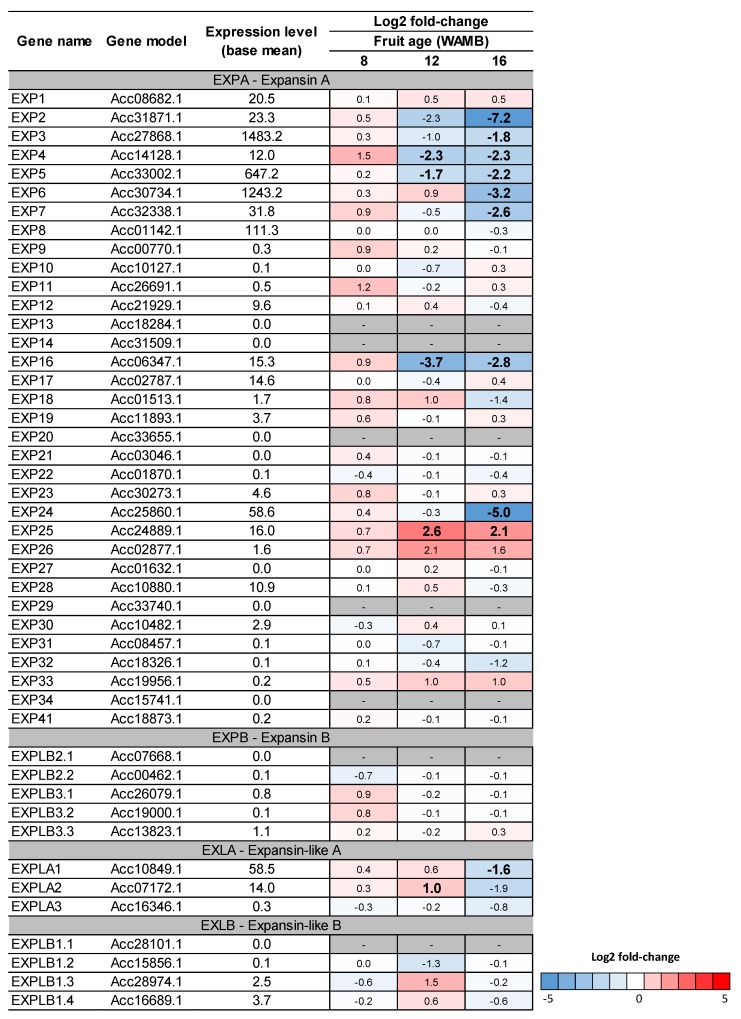
Heat-map of kiwifruit expansin genes. DESeq comparison of low carbohydrate supply (carbon starvation) versus high carbohydrate supply treatments. Differentially expressed genes were identified based on the DESeq analysis from Nardozza et al. [6]. For each gene, the expression level (base mean) and the log2 fold-change are presented. Fruit age is in weeks after mid bloom (WAMB). Bold figures mean differences are significant for adjusted *p* < 0.05 (DESeq analysis).

Expansins are a class of cell wall proteins able to drive non-enzymatic pH-dependent cell wall relaxation by partnering with H(+)-ATPAses proton pumps [16,33]. Of the expansin genes identified in kiwifruit [30], the expansin A class genes were the most affected by carbon starvation at 16 WAMB. *EXP2*-*7* and *EXP24* were significantly downregulated under carbon starvation (Figure 8). Highly expressed *EXP3*, *EXP5*, and *EXP6* were downregulated by 3.5-, 4.5-, and 9-fold, respectively. In addition, plasma membrane H(+)-ATPase genes were also downregulated (Figure S4). 

## 3. Discussion

In kiwifruit, fruit growth increases with an increase in carbohydrate supply [4,5]. The fruit expansion phase in kiwifruit appears to differ from those of other fleshy fruits. Auxin and/or gibberellins drive fruit expansion in strawberry [34], apple [10], and tomato [35]. Our results showed that cytokinins are key phytohormones during fruit expansion in kiwifruit. Phytohormone profiling revealed that fruit weight differences obtained under contrasting carbohydrate supplies positively correlated with cytokinin concentrations in the fruit outer pericarp. Changes in gibberellin, jasmonic acid, and abscisic acid concentrations were mostly developmental in nature and not strongly affected by carbohydrate supply. Interestingly, gibberellin precursor GA19 increased under carbon starvation, however, this effect was not maintained downstream in the biosynthetic pathway, and both GA20 and active GA1 concentrations were unaffected, suggesting that the role of gibberellin was not significant in the kiwifruit cell expansion phase. Surprisingly, unconjugated auxin concentrations were not correlated with fruit growth and were also not affected by fruit carbohydrate supply. This is in contrast to observations in fleshy fruit types [10,34,35], suggesting that free auxin is not critical during the fruit expansion phase of kiwifruit. However, we cannot rule out that conjugated auxins, which were not measured in this study, may play a role.

Similar to Arabidopsis seedlings, where de novo cytokinin synthesis is induced by sugar, our data showed that in carbon-starved kiwifruit, cytokinin synthesis is repressed. This is via downregulation of the cytokinin biosynthetic genes *IPTs* and *CYP735As*, suggesting that sugar control over cytokinin production could apply to sink tissues. Signalling of carbon starvation in kiwifruit is mediated by the sucrose non-fermenting-1 related protein kinase 1 via trehalose 6-phosphate [6]. In Arabidopsis, it has been proposed that cytokinins have a role in regulating the genes of the trehalose 6-phosphate pathway [17]. A similar scenario may occur in kiwifruit outer pericarp tissue, where the observed reduction of trehalose 6-phosphate concentration in carbon-starved fruit [6] could be triggered by cytokinin signalling.

In maturing kiwifruit, cytokinin catabolism was not critical for controlling cytokinin concentrations [15], whilst in other plant species (e.g., cabbage, maize, and wheat) it has been suggested that *IPT* and *CKX* transcription levels are coupled [31,36,37] and CKX is a key enzyme for cytokinin homeostasis. Our results show that genes coding for cytokinin catabolic enzymes were only marginally affected by carbon supply treatments and cytokinin dehydrogenase was not associated with different cytokinin concentrations (Figure 2; Figure 7). These data suggest that, in kiwifruit, carbon starvation affects de novo synthesis rather than irreversible cleavage of cytokinins during the fruit expansion phase.

Whilst transcriptional data for cytokinin biosynthetic genes supported the phytohormone data, the regulation of the multistep phosphorelay cytokinin signalling was less clear. Under carbon starvation, we observed an upregulation of the genes involved in the multistep phosphorelay pathway (histidine kinase, histidine phosphotransferase, and type-B RRs), suggesting that they might not be controlled at the transcriptional level as transcription is triggered by decreased cytokinin concentrations. Multistep phosphorelay signalling is triggered by cytokinin binding, which leads to autophosphorylation of histidine kinase. The phosphate is then transferred to histidine phosphotransferase and the final acceptor type-B RR proteins, which positively regulate transcription in the nucleus (reviewed by Kieber and Schaller [38]). In contrast, type-A RRs are negative feedback regulators in cytokinin signalling and are transcriptionally activated by type-B RRs [39]. In kiwifruit outer pericarp, type-A RRs were mostly downregulated when cytokinin concentrations were low. This is in agreement with findings from Bhargava et al. [40], where type-A response regulators were upregulated in response to exogenous cytokinin applications, suggesting an increase in endogenous cytokinins could lead to a similar response.

Expansins are proteins involved in the acidic relaxation of plant cell walls and drivers of short-term cell expansion [41]. Effects of cytokinins on expansin-mediated cell wall relaxation and cell expansion has been suggested [42]. A meta-analysis of cytokinin effects on Arabidopsis transcriptome identified expansins as targets of cytokinin signalling [43]. Expansins drive acidic cell elongation in Arabidopsis roots, where α-expansinA genes and plasma membrane H(+)-ATPase are controlled by cytokinins via ARR1 [44]. In soybean, cytokinins regulate the expression of expansin and cell wall expansion [45]. The effects of cytokinins on expansin during cell expansion and fruit expansion have not been well characterised. A study of grape berries associated cytokinin concentrations with post-veraison cell expansion and berry growth [19]. Exogenous cytokinin application induced expansin expression in white sweet clover (*Melilotus alba*) [42]. In kiwifruit, under carbon starvation, we observed significant decreases in cytokinin concentrations, cytokinin biosynthetic genes transcription, a-expansin transcription, and fruit weight, suggesting a role for expansin in driving cell expansion and fruit expansion. Cytokinin concentrations were developmentally driven as the positive correlation between total cytokinin concentration and fruit weight held true also within each treatment, in agreement with Pilkington et al. [15]. Further experimentation will be required to clarify the mechanism and if, similar to Arabidopsis roots, the signalling may be mediated by a type-B RR [44].

In conclusion, our results suggest a new role for cytokinins in kiwifruit growth, where they contribute to stimulate cell expansion via a mechanism that could involve expansin protein. This is in contrast to other fleshy fruits where cytokinins are a key phytohormone class during cell division, and further supports the responsiveness of kiwifruit to exogenous cytokinin treatments following the cell division phase. We acknowledge a limitation of this study in discussing the functional role of cytokinin in kiwifruit as developmental phases were inferred from the literature [1,2], rather than defined from direct cell number and cell size observations. Overall, these findings contribute to expand the current knowledge on fruit weight determination in kiwifruit. 

## 4. Materials and Methods 

### 4.1. Plant Material

*Actinidia chinensis* (Planch.) var. *chinensis* ‘Zes006′ (red fleshed) kiwifruit was sampled as per the experiments described in Nardozza et al. [6]. The outer pericarp tissue from the following treatments was used: high carbohydrate supply (girdled shoot with leaf to fruit ratio of 4), low carbohydrate supply (girdled shoot with a leaf to fruit ratio of 1), and untreated control (ungirdled shoot with a leaf to fruit ratio of 1; vine standard [5]). Individual shoots represented the experimental unit. Girdles were applied to shoots at 4 WAMB and maintained open until the end of the experiment. Only samples collected at 8, 12, and 16 WAMB were considered to match the transcriptomic data. Fresh fruit weight data are given in Nardozza et al. [6]. Outer pericarp tissues were collected to include three to four biological replicates (due to lost samples in the field or during analysis; see details in Table S2), snap frozen in liquid nitrogen, and stored at –80 °C until further analysis.

### 4.2. Phytohormones

#### 4.2.1. Phytohormone Extraction and Fractionation

Frozen plant material was ground in liquid nitrogen to a fine powder using a mortar and pestle and stored at –80 °C for chemical analysis. To each sample (200 mg fresh weight), 1 mL chilled (4 °C) extraction solvent 80:20 CH_3_CN:H_2_O, labelled internal standard mix ([^2^H_5_] Z 10 ng, [^2^H_5_] (9R)Z 3 ng, [^2^H_5_] t-ZROG 30 ng, [^2^H_5_] (9G)Z 3 ng, [^2^H_6_] iP 0.5 ng, [^2^H_6_] (9R) iP 1 ng, [^2^H_6_] iP9G 3 ng, [^2^H_5_] t-ZOG 7 ng, [^2^H_2_] GA7 0.5 ng, [^2^H_4_] SA 1 ng, [^2^H_6_] ABA 2 ng, [^2^H_5_] JA 2 ng, [^2^H_10_] JA-Ile 2 ng, [^13^C_6_] IAA 10 ng; OlchemIm Ltd., Olomouc, Czech Republic), and 0.8 g stainless steel beads 0.9–2 mm (Next Advance Inc., Troy, NY, USA) were added. Samples were bead beaten for 5 min (Bullet Blender 24 Gold, Next Advance Inc., Troy, NY, USA) and then extracted overnight in the dark at 4 °C using an end-over-end rotator at 30 rotations/min. After centrifugation at 13,000× *g* for 5 min, the supernatant was transferred into a 96-well collection plate (Phenomenex, Torrance, CA, USA). The remaining pellet was re-extracted twice with 1 mL 80:20 CH_3_CN:H_2_O overnight, and combined with the first supernatant, and evaporated to dryness using a CentriVap concentrator (Labcon, Petaluma, CA, USA). Samples were reconstituted in 1 mL 1 M formic acid (aq) and placed on an orbital shaker at room temperature for 1 h at 230 rpm (IKA Labortechnik, Staufen, Germany). To remove interfering compounds, the extract was passed through a SOLA SCX 96-well plate (10 mg/2 mL, Thermo Scientific) equilibrated with 4 mL acetonitrile and conditioned with 4 mL 1 M formic acid (aq). After conditioning, the reconstituted samples were loaded, washed with 3 mL of 1 M formic acid (aq), 3 mL of water, and the acidic plant hormones were eluted with 1.5 mL acetonitrile (Fraction A). The plate was then washed with 1 mL water, followed by 1.5 mL of 0.35 M ammonium hydroxide and the cytokinins were eluted with 0.5 mL of 0.35 M ammonium hydroxide in 60% acetonitrile (Fraction B). Each fraction was evaporated to dryness using a CentriVap concentrator (Labcon, Petaluma, CA, USA).

Prior to derivatisation with bromocholine bromide (BETA), Fraction A was further purified using a modification of a method described by Kojima et al. [46] to increase the sensitivity of analysis for gibberellins. Briefly, samples were reconstituted in 1 mL 80:20 CH_3_CN:H_2_O + 4% trimethylamine and applied to a Hypersep 96-well plate (Hypercarb 25 mg/1 mL, Thermo Scientific) equilibrated with 1 mL acetonitrile and conditioned with 1 mL water. The sample eluate was collected into a 96-well collection plate and a further 0.5 mL CH_3_CN + 4% trimethylamine was applied to the Hypersep plate and collected into the same collection plate. After evaporation, the samples were reconstituted with 160 µL CH_3_CN and to each sample 40 µL of derivatisation solution (6.25 M BETA in 86% acetonitrile) and 20 µL 4% trimethylamine in CH_3_CN was added. The mixed solution was incubated at 50 °C at 300 rpm for 24 h (Eppendorf, ThermoMixer C, Hamburg, Germany) and then evaporated to dryness.

#### 4.2.2. Liquid-Chromatography Tandem Mass-Spectrometry (LC-MS/MS) Analysis 

Liquid-Chromatography Tandem Mass-Spectrometry (LC-MS/MS) experiments were performed on a 5500 QTrap triple quadrupole/linear ion trap (QqLIT) mass spectrometer equipped with a TurboIon-Spray^TM^ interface (AB Sciex, Concord, ON, Canada) coupled to an Ultimate 3000 UHPLC (Dionex, Sunnyvale, CA, USA). 

#### 4.2.3. Cytokinins

For cytokinins, dried samples from Fraction B were reconstituted in 200 µL 10:90 CH_3_OH:H_2_O + 1% acetic acid and filtered through a conditioned (200 µL CH_3_OH) 96-well 0.45 µm hydrophobic filter plate (Pall Filters, AcroPrep, Cortland, NY, USA) prior to mass spectrometric (MS) analysis. Cytokinins were separated on an Acquity UPLC BEH C18 1.7 µm 2.1 × 150 mm ID column (Waters, Wexford, Ireland) maintained at 70 °C. Solvents were (A) 15 mM ammonium formate adjusted to pH 4 with formic acid and (B) methanol with a flow rate of 350 μL min^−1^. The initial mobile phase, 15% B was held for 6.5 min, then ramped linearly to 20% B at 9 min, then to 50% B at 12.5 min and 100% B at 14 min and holding at 100% B for 1 min before resetting to the original conditions. Injection size was 10 µL. MS data were acquired in the positive ion mode using a scheduled multiple reaction monitoring (MRM) method. The transitions monitored (Q1 and Q3) are listed in Table S3. Other operating parameters were as follows: ion spray voltage 4500 V; temperature 600 °C; curtain gas 45 psi; ion source gas 1 60 psi; ion source gas 2 60 psi; collision gas set to medium.

#### 4.2.4. Acidic Phytohormones

For acidic phytohormones (gibberellins, auxins, jasmonates, salicylates and abscisates), Fraction A dried and derivatised samples were reconstituted in 100 µL 5% (CH_3_OH:CH_3_CN): 95% (5 mM ammonium formate adjusted to pH 3.7 with formic acid). An internal standard for each analyte was created by derivatising a mixed analytical standard with a deuterated analogue of bromocholine bromide ([^2^H_9_]-BETA) using a modified method to that described by Sun et al. [47], as here detailed. To each sample, 100 µL of internal standard (5 ppb [^2^H_9_]-BETA) was added. Samples were filtered through a 0.7-µm glass filter plate prior to LC-MS analysis. Acidic phytohormones were separated on an Acquity UPLC BEH C18 1.7 µm 2.1 × 150 mm ID column (Waters, Wexford, Ireland) maintained at 40 °C. Solvents were (A) 5% (CH_3_OH:CH_3_CN):95% (5 mM ammonium formate adjusted to pH 3.7 with formic acid) and (B) 95% (CH_3_OH:CH_3_CN):5% (5 mM ammonium formate adjusted to pH 3.7 with formic acid) with a flow rate of 250 μL min^−1^. The initial mobile phase, 0% B was held for 1 min before ramping linearly to 5.3% B at 2 min, 7.5% B at 5.5 min, 40% B at 11 min, and holding for 3.5 min before ramping to 100% B at 15.5 min and holding at 100% B until 21 min before resetting to the original conditions. Injection size was 2 µL. MS data were acquired in the positive mode using a scheduled MRM method. The transitions monitored (Q1 and Q3) are listed in Table S4. Transitions for compounds other than gibberellins were detuned from optimum to reduce their sensitivity to fit within the dynamic linear range of the instrument. Other operating parameters were as follows: ion spray voltage 4500 V; temperature 600 °C; curtain gas 45 psi; ion source gas 1 60 psi; ion source gas 2 60 psi; collision gas set to medium.

#### 4.2.5. Phytohormone Identification and Quantification

Identification and quantification of all compounds were confirmed through the comparison of the acquired spectra with spectra from the authentic standards. All data were analysed and processed using Analyst version 1.6.2 and MultiQuant version 3.0 software packages. Concentrations were calculated for each compound in equivalence to their respective stable isotope as the internal standard.

### 4.3. Transcriptomic Data

The Red5 *A. chinensis* var. *chinensis* genome [30] was mined for the gene models involved in the cytokinin biosynthetic, catabolic, and signalling pathways, and for plasma membrane H(+)-ATPase genes. The expansin gene models list was sourced from Pilkington et al. [30]. Gene models were then searched in the differentially expressed gene (DEG) lists generated by Nardozza et al. [6] (Bioproject ID PRJNA593615) and heat maps were created to visualise the effect of the carbohydrate supply on gene transcription, with a focus on the two girdled treatments. DESeq output is shown in Figure S5. 

### 4.4. Statistical Analysis

The effect of the carbohydrate supply and fruit age (factors) on fruit weight and phytohormone concentrations were analysed using a linear mixed effects model (LME, type 3 sums of squares Kenward–Roger’s method) in R (version 3.5.1) [48]. The biological replicates were treated as random effects. When significant effects or interactions were present, the means were separated on the basis of all pairwise comparisons of least-squares means, adjusted for multiple comparison by Tukey’s correction (letters assigned; confidence level 95%). Residual plots were inspected to check for the assumptions of normality and constant variance. Where appropriate, a log-transformation was used prior to analysis with the fitted means back-transformed onto the original scale. Pearson correlation analysis was performed to identify positive or negative correlations between fruit weight and phytohormone concentration. Principal component analysis for cytokinin hormones were also performed in R using the MixOmics libraries [49]. Data were plotted using package ggplot2 [50] with 95% confidence interval ellipses.

## Supplementary Materials

The following are available online at https://www.mdpi.com/2218-1989/10/1/23/s1. Figure S1: Cytokinin biplot at 16 weeks after mid bloom; Figure S2: Heat-map for multistep phosphorelay (MSP) pathway kiwifruit genes: cytokinin receptors histidine kinases and positive regulators histidine-containing phosphotransfer proteins; Figure S3: Heat-map for multistep phosphorelay (MSP) pathway kiwifruit genes: negative regulators A-type Arabidopsis Response Regulators (ARR) and positive regulators B-type ARR; Figure S4: Heat-map of kiwifruit plasma membrane H(+)-ATPase genes; Table S1: Proportion of cytokinins types on total cytokinin concentrations in different treatments; Table S2: List of collected samples and biological replicates used for the experiments; Table S3: Multiple reaction monitoring (MRM) transitions used for cytokinin analysis; Table S4: Multiple reaction monitoring (MRM) transitions used for acidic phytohormone analysis; Table S5: DESeq pairwise comparison between low carbohydrate supply (LC) and high carbohydrate supply (HC) samples at 8, 12 and 16 weeks after mid bloom.

## References

[1] Gould K., Ferguson I.B., Atwell B.J., Kriedeman P.E., Turnbull C.G.N. (2010). Kiwifruit development: A case study. Plants in Action: Adaptation in Nature, Performance in Cultivation.

[2] Nardozza S., Boldingh H.L., Osorio S., Hohne M., Wohlers M., Gleave A.P., MacRae E.A., Richardson A.C., Atkinson R.G., Sulpice R. (2013). Metabolic analysis of kiwifruit (*Actinidia deliciosa*) berries from extreme genotypes reveals hallmarks for fruit starch metabolism. J. Exp. Bot..

[3] Richardson A., Boldingh H., McAtee P., Gunaseelan K., Luo Z., Atkinson R., David K., Burdon J., Schaffer R. (2011). Fruit development of the diploid kiwifruit, *Actinidia chinensis* ‘Hort16A’. BMC Plant Biol..

[4] Lai R., Woolley D., Lawes G.S. (1989). Effect of leaf:fruit ratio on fruit growth of kiwifruit (*Actinidia deliciosa*). Sci. Hortic..

[5] Minchin P.E.H., Snelgar W.P., Blattmann P., Hall A.J. (2010). Competition between fruit and vegetative growth in Hayward kiwifruit. N. Z. J. Crop Hortic. Sci..

[6] Nardozza S., Boldingh H.L., Kashuba P., Feil R., Jones D., Thrimawithana A.H., Ireland H.S., Philippe M., Wohlers M.W., McGhie T. (2019). Carbon starvation reduces carbohydrate and anthocyanin accumulation in red-fleshed fruit via trehalose 6-phosphate and MYB27. Plant Cell Environ..

[7] Nardozza S., Boldingh H., Kashuba M., McCaughan L., Philippe M., Wohlers M., McGhie T., Currie M., Montefiori M., Richardson A. (2017). Effects of the manipulation of carbohydrate supply on fruit dry matter and colour development in a block-red *Actinidia chinensis* var. chinensis genotype. Acta Hortic..

[8] McAtee P., Karim S., Schaffer R., David K. (2013). A dynamic interplay between phytohormones is required for fruit development, maturation, and ripening. Front. Plant Sci..

[9] Kumar R., Khurana A., Sharma A.K. (2014). Role of plant hormones and their interplay in development and ripening of fleshy fruits. J. Exp. Bot..

[10] Devoghalaere F., Doucen T., Guitton B., Keeling J., Payne W., Ling T.J., Ross J.J., Hallett I.C., Gunaseelan K., Dayatilake G.A. (2012). A genomics approach to understanding the role of auxin in apple (*Malus x domestica*) fruit size control. BMC Plant Biol..

[11] Giovannoni J.J. (2004). Genetic regulation of fruit development and ripening. Plant Cell.

[12] Nardozza S., Boldingh H.L., Wohlers M.W., Gleave A.P., Luo Z., Costa G., MacRae E.A., Clearwater M.J., Richardson A.C. (2017). Exogenous cytokinin application to *Actinidia chinensis* var. *deliciosa* ‘Hayward’ fruit promotes fruit expansion through water uptake. Hortic. Res..

[13] Hopping M.E. (1976). Effect of exogenous auxins, gibberellins, and cytokinins on fruit development in Chinese gooseberry (*Actinidia chinensis* Planch.). N. Z. J. Bot..

[14] Lewis D.H., Burge G.K., Schmierer D.M., Jameson P.E. (1996). Cytokinins and fruit development in the kiwifruit (*Actinidia deliciosa*). 1. Changes during fruit development. Physiol. Plant..

[15] Pilkington S.M., Montefiori M., Galer A.L., Emery R.J.N., Allan A.C., Jameson P.E. (2013). Endogenous cytokinin in developing kiwifruit is implicated in maintaining fruit flesh chlorophyll levels. Ann. Bot..

[16] Cosgrove D.J. (2005). Growth of the plant cell wall. Nat. Rev. Mol. Cell Biol..

[17] Brenner W.G., Ramireddy E., Heyl A., Schmulling T. (2012). Gene regulation by cytokinin in Arabidopsis. Front. Plant Sci..

[18] Ljung K., Nemhauser J.L., Perata P. (2015). New mechanistic links between sugar and hormone signalling networks. Curr. Opin. Plant Biol..

[19] Bottcher C., Burbidge C.A., Boss P.K., Davies C. (2015). Changes in transcription of cytokinin metabolism and signalling genes in grape (*Vitis vinifera* L.) berries are associated with the ripening-related increase in isopentenyladenine. BMC Plant Biol..

[20] Argueso C.T., Ferreira F.J., Kieber J.J. (2009). Environmental perception avenues: The interaction of cytokinin and environmental response pathways. Plant Cell Environ..

[21] Roitsch T., Ehneß R. (2000). Regulation of source/sink relations by cytokinins. Plant Growth Regul..

[22] Werner T., Holst K., Pors Y., Guivarc’h A., Mustroph A., Chriqui D., Grimm B., Schmulling T. (2008). Cytokinin deficiency causes distinct changes of sink and source parameters in tobacco shoots and roots. J. Exp. Bot..

[23] Kiba T., Takebayashi Y., Kojima M., Sakakibara H. (2019). Sugar-induced de novo cytokinin biosynthesis contributes to Arabidopsis growth under elevated CO_2_. Sci. Rep..

[24] Hwang I., Sheen J. (2001). Two-component circuitry in Arabidopsis cytokinin signal transduction. Nature.

[25] Ma J.K., Hanssen M., Lundgren K., Hernandez L., Delatte T., Ehlert A., Liu C.M., Schluepmann H., Droge-Laser W., Moritz T. (2011). The sucrose-regulated Arabidopsis transcription factor bZIP11 reprograms metabolism and regulates trehalose metabolism. New Phytol..

[26] Nardozza S., Hallett I.C., McCartney R., Richardson A.C., MacRae E.A., Costa G., Clearwater M.J. (2011). Is fruit anatomy involved in variation in fruit starch concentration between *Actinidia deliciosa* genotypes?. Funct. Plant Biol..

[27] Richardson A., Boldingh H., Kashuba P., Knight G., Ellingham D. (2019). Flowering time determines the weight and composition of *Actinidia chinensis* var. chinensis ‘Zesy002′ kiwifruit. Sci. Hortic..

[28] Hirose N., Takei K., Kuroha T., Kamada-Nobusada T., Hayashi H., Sakakibara H. (2008). Regulation of cytokinin biosynthesis, compartmentalization and translocation. J. Exp. Bot..

[29] Sakakibara H. (2006). Cytokinins: Activity, biosynthesis, and translocation. Annu. Rev. Plant Biol..

[30] Pilkington S.M., Crowhurst R., Hilario E., Nardozza S., Fraser L., Peng Y., Gunaseelan K., Simpson R., Tahir J., Deroles S.C. (2018). A manually annotated *Actinidia chinensis* var. *chinensis* (kiwifruit) genome highlights the challenges associated with draft genomes and gene prediction in plants. BMC Genom..

[31] Song J.C., Jiang L.J., Jameson P.E. (2012). Co-ordinate regulation of cytokinin gene family members during flag leaf and reproductive development in wheat. BMC Plant Biol..

[32] Kieber J.J., Schaller G.E. (2014). Cytokinins. Arab. book.

[33] Marowa P., Ding A.M., Kong Y.Z. (2016). Expansins: Roles in plant growth and potential applications in crop improvement. Plant Cell Rep..

[34] Csukasi F., Osorio S., Gutierrez J.R., Kitamura J., Giavalisco P., Nakajima M., Fernie A.R., Rathjen J.P., Botella M.A., Valpuesta V. (2011). Gibberellin biosynthesis and signalling during development of the strawberry receptacle. New Phytol..

[35] de Jong M., Wolters-Arts M., García-Martínez J.L., Mariani C., Vriezen W.H. (2010). The *Solanum lycopersicum* AUXIN RESPONSE FACTOR 7 (*SlARF7*) mediates cross-talk between auxin and gibberellin signalling during tomato fruit set and development. J. Exp. Bot..

[36] Liu Z.N., Lv Y.X., Zhang M., Liu Y.P., Kong L.J., Zou M.H., Lu G., Cao J.S., Yu X.L. (2013). Identification, expression, and comparative genomic analysis of the IPT and CKX gene families in Chinese cabbage (*Brassica rapa* ssp *pekinensis*). BMC Genom..

[37] Brugiere N., Jiao S.P., Hantke S., Zinselmeier C., Roessler J.A., Niu X.M., Jones R.J., Habben J.E. (2003). Cytokinin oxidase gene expression in maize is localized to the vasculature, and is induced by cytokinins, abscisic acid, and abiotic stress. Plant Physiol..

[38] Kieber J.J., Schaller G.E. (2018). Cytokinin signaling in plant development. Development.

[39] Taniguchi M., Sasaki N., Tsuge T., Aoyama T., Oka A. (2007). ARR1 directly activates cytokinin response genes that encode proteins with diverse regulatory functions. Plant Cell Physiol..

[40] Bhargava A., Clabaugh I., To J.P., Maxwell B.B., Chiang Y.-H., Schaller G.E., Loraine A., Kieber J.J. (2013). Identification of cytokinin-responsive genes using microarray meta-analysis and RNA-Seq in Arabidopsis. Plant Physiol..

[41] Cosgrove D.J. (2000). Loosening of plant cell walls by expansins. Nature.

[42] Lee A., Giordano W., Hirsch A.M. (2008). Cytokinin induces expansin gene expression in *Melilotus alba* Desr. wild-type and the non-nodulating, non-mycorrhizal (NodMyc) mutant Masym3. Plant Signal. Behav..

[43] Brenner W.G., Schmulling T. (2015). Summarizing and exploring data of a decade of cytokinin-related transcriptomics. Front. Plant Sci..

[44] Pacifici E., Di Mambro R., Dello Ioio R., Costantino P., Sabatini S. (2018). Acidic cell elongation drives cell differentiation in the Arabidopsis root. EMBO J..

[45] Downes B.P., Crowell D.N. (1998). Cytokinin regulates the expression of a soybean beta-expansin gene by a post-transcriptional mechanism. Plant Mol. Biol..

[46] Kojima M., Kamada-Nobusada T., Komatsu H., Takei K., Kuroha T., Mizutani M., Ashikari M., Ueguchi-Tanaka M., Matsuoka M., Suzuki K. (2009). Highly sensitive and high-throughput analysis of plant hormones using MS-probe modification and Liquid Chromatography—Tandem Mass Spectrometry: An application for hormone profiling in *Oryza sativa*. Plant Cell Physiol..

[47] Sun X.H., Ouyang Y., Chu J.F., Yan J., Yu Y., Li X.Q., Yang J., Yan C.Y. (2014). An in-advance stable isotope labeling strategy for relative analysis of multiple acidic plant hormones in sub-milligram Arabidopsis thaliana seedling and a single seed. J. Chromatogr..

[48] R Core Team (2018). R: A Language and Environment for Statistical Computing.

[49] Rohart F., Gautier B., Singh A., Lê Cao K.-A. (2017). mixOmics: An R package for ‘omics feature selection and multiple data integration. PLoS Comp. Biol..

[50] Wickham H. (2016). Ggplot2: Elegant graphics for Data Analysis.

