# Supplementary material for: Phytohormone and Transcriptomic Analysis Reveals Endogenous Cytokinins Affect Kiwifruit Growth under Restricted Carbon Supply
Source: Metabolites. 2020 Jan 4;10(1):23. doi: 10.3390/metabo10010023 (PMC7022440; doi:10.3390/metabo10010023)
Supplement: Supplementary file 1 [file metabolites-10-00023-s001.zip › Supplementary figures_SN.pdf]

Supplementary Figures

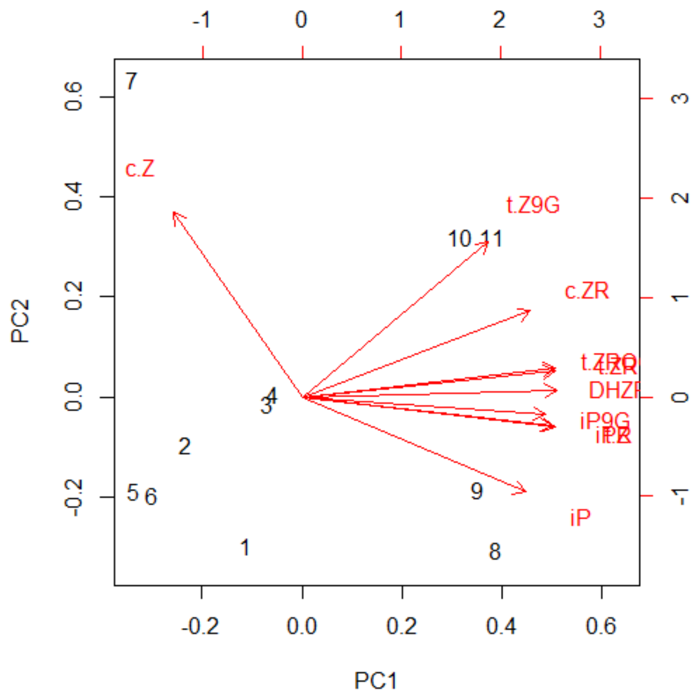

Figure S1: Cytokinin biplot at 16 weeks after mid bloom. Numbers 1-3 are low carbohydrate supply kiwifruit, numbers 4-7 are untreated control fruit, and numbers 8-11 are high carbohydrate supply fruit.

Gene name	Gene model	Expression level (base mean)	Log2 fold-change Fruit age (WAMB)		
			8	12	16
Histidine kinase					
AHK2.1	Acc14922.1	419.3	0.2	0.3	**1.0**
AHK3.1	Acc11804.1	47.0	0.2	-0.3	0.3
AHK3.2	Acc11805.1	140.9	0.0	-0.1	**0.4**
AHK3.3	Acc14294.1	676.8	0.1	0.2	**0.3**
AHK3.4	Acc32738.1	690.1	0.1	**0.4**	**0.3**
AHK3.5	Acc06165.1	533.3	0.1	**0.4**	**0.9**
AHK4.1	Acc28323.1	65.7	0.2	-0.1	0.3
AHK4.2	Acc08110.1	59.1	0.0	0.3	**0.7**
AHK4.3	Acc08111.1	34.9	0.1	-0.6	**0.7**
Histidine-containing phosphotransfer protein					
AHP1.1	Acc15213.1	104.9	-0.2	0.1	-0.2
AHP1.2	Acc17231.1	185.4	0.1	**0.9**	0.1
AHP1.3	Acc19586.1	41.2	0.0	-0.1	-0.4
AHP1.4	Acc27271.1	93.1	-0.1	0.0	**-0.3**
AHP1.5	Acc31641.1	64.7	-0.1	0.0	-0.1
AHP1.6	Acc04324.1	39.2	-0.1	0.2	0.3
AHP1.7	Acc09459.1	252.2	-0.1	**1.5**	**2.3**
AHP4.1	Acc12680.1	0.8	0.7	0.6	0.3
AHP4.2	Acc17743.1	0.5	-1.0	0.2	-0.8
AHP4.3	Acc28626.1	12.5	0.2	0.8	0.4

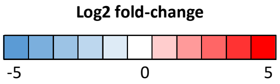

Figure S2: Heat-map of kiwifruit multistep phosphorelay (MSP) pathway genes: cytokinin receptors histidine kinases (AHK) and positive regulators histidine-containing phosphotransfer proteins (AHP). DESeq comparison of low carbohydrate supply versus high carbohydrate supply treatments. For each gene, the expression level (base mean) and the log2 fold-change are presented. Fruit age is in weeks after mid bloom (WAMB). Bold figures mean differences are significant for adjusted *p*<0.05.

Gene name	Gene model	Expression level (base mean)	Log2 fold-change Fruit age (WAMB)		
			8	12	16
Type-A ARR					
ARR3.1	Acc17017.1	1.3	0.4	-1.6	-1.2
ARR4.1	Acc06265.1	112.5	-0.1	**-1.2**	-0.5
ARR6.1	Acc18177.1	126.1	0.2	-0.7	-0.2
ARR6.2	Acc29817.1	6.0	0.6	-1.6	**-1.7**
ARR9.1	Acc13210.1	13.7	0.2	-0.7	0.2
ARR9.2	Acc23103.1	78.7	0.1	-0.7	-0.3
ARR9.3	Acc24383.1	0.7	-0.6	-0.7	-2.5
ARR9.4	Acc03913.1	1.0	-0.4	**-2.9**	-1.6
ARR9.5	Acc05075.1	6.9	0.7	-0.5	**-1.0**
ARR9.6	Acc09239.1	0.3	-0.7	0.6	-1.5
ARR17.1	Acc16581.1	0.0	-	-	-
ARR17.2	Acc02781.1	0.3	-0.8	0.6	-0.1
Type-B ARR					
ARR1.1	Acc13982.1	87.2	0.3	-0.2	0.1
ARR2.1	Acc24578.1	84.3	-0.1	-0.1	**0.3**
ARR2.2	Acc27152.1	242.5	0.0	-0.3	**-0.5**
ARR2.3	Acc28375.1	498.6	0.0	-0.2	0.0
ARR2.4	Acc29737.1	5.0	-0.4	-0.6	0.1
ARR2.5	Acc31143.1	223.9	0.1	**0.2**	0.1
ARR2.6	Acc32853.1	117.4	0.0	0.0	0.2
ARR2.7	Acc03501.1	96.6	-0.1	0.2	**0.4**
ARR10.1	Acc00754.1	12.2	0.2	1.2	0.6
ARR10.2	Acc10062.1	0.7	1.5	-0.1	0.9
ARR10.3	Acc10115.1	0.4	-0.2	1.9	1.3
ARR11.1	Acc08222.1	11.4	0.0	0.3	**1.0**
ARR11.2	Acc33711.1	28.3	-0.3	0.6	0.6
ARR12.1	Acc00691.1	6.8	0.1	0.7	0.4
ARR12.2	Acc12420.1	45.2	-0.1	0.0	**-0.5**
ARR12.3	Acc14541.1	78.0	0.0	0.0	0.3
ARR12.4	Acc18510.1	8.8	-0.3	-0.1	0.2
ARR12.5	Acc29214.1	116.0	0.0	**0.8**	**0.7**
ARR12.6	Acc09091.1	14.0	0.3	0.4	0.7

Log2 fold-change

-5 0 5

Figure S3: Heat-map of kiwifruit multistep phosphorelay (MSP) pathway genes: negative regulators A-type Arabidopsis Response Regulators (Type-A ARR) and positive regulators B-type ARR (Type-B ARR). DESeq comparison of low carbohydrate supply versus high carbohydrate supply treatments. For each gene, the expression level (base mean) and the log2 fold-change are presented. Fruit age is in weeks after mid bloom (WAMB). Bold figures mean differences are significant for adjusted $p<0.05$.

Gene name	Gene model	Expression level (base mean)	Log2 fold-change		
			Fruit age (WAMB)		
			8	12	16
Plasma membrane H(+)-ATPase					
AHA1.1	Acc00906.1	516.9	-0.1	**-0.8**	**-0.9**
AHA2.1	Acc09795.1	1516.8	-0.1	**-0.4**	**0.3**
AHA2.2	Acc22690.1	6.6	-0.6	-0.7	-0.1
AHA7.1	Acc07688.1	0.8	0.4	-0.1	0.9
AHA7.2	Acc14064.1	375.4	-0.1	0.2	**0.9**
AHA8.1	Acc00689.1	29.3	0.0	-0.6	**-1.1**
AHA8.2	Acc10061.1	0.8	-2.1	0.2	1.7
AHA8.3	Acc14537.1	10.5	-0.5	-0.2	-0.6
AHA9.1	Acc22147.1	0.2	0.0	0.0	0.3
AHA9.2	Acc26895.1	0.2	0.1	-0.2	-0.1
AHA10.1	Acc12867.1	59.2	-0.1	-0.6	**-1.4**
AHA10.2	Acc17609.1	801.3	-0.1	-0.1	**-0.7**
AHA11.1	Acc02371.1	180.5	-0.6	**-0.8**	**-1.0**
AHA11.2	Acc15848.1	54.3	-0.2	-0.2	-0.1
AHA11.3	Acc16129.1	0.1	0.9	0.2	0.6
AHA11.4	Acc18223.1	1390.9	0.0	-0.1	**0.3**
AHA11.5	Acc29838.1	2399.3	0.0	-0.3	-0.1

Log2 fold-change

-5 0 5

Figure S4: Heat-map of kiwifruit plasma membrane H(+)-ATPase genes. DESeq comparison of low carbohydrate supply versus high carbohydrate supply treatments. For each gene, the expression level (base mean) and the log2 fold-change are presented. Fruit age is in weeks after mid bloom (WAMB). Bold figures mean differences are significant for adjusted $p<0.05$.
